# Relative Preference of Cannabis, Alcohol, and Nicotine Products Among U.S. Adolescents and Young Adults Who Are Using or Susceptible to Cannabis Use: Insights from a Best-Worst Scaling Study

**DOI:** 10.1080/10826084.2026.2731166

**Published:** 2026-09-22

**Authors:** Lei Xu, Shiqi Zhang, Mohamed Elsaid, Ce Shang

**Affiliations:** a Division of Medical Oncology, Department of Internal Medicine, College of Medicine, The Ohio State University Wexner Medical Center, Columbus, Ohio, USA; b Center for Tobacco Research, The Ohio State University Comprehensive Cancer Center, The Ohio State University, Columbus, Ohio, USA; c Division of Biostatistics and Population Health, Department of Biomedical Informatics, College of Medicine, The Ohio State University Wexner Medical Center, Columbus, Ohio, USA

**Keywords:** Adolescents, addiction/dependence, substance use comorbidities, cannabis/marijuana/cannabinoid, nicotine reduction, social determinants

## Abstract

**Background:**

The prevalence of multiple substance use is high among U.S. adolescents and young adults (AYAs), particularly as market shifts with alcohol and cannabis being the most prevalent substances and nicotine vaping displacing cigarette smoking among this population. However, it remains unclear how AYAs evaluate diverse products in terms of preference, addictiveness, and risk.

**Methods:**

We analyzed data from a nationally representative sample of 1,010 U.S. AYAs (aged 15–21) in June 2024 who had used cannabis in the past 30 days or were susceptible to use. Best-worst scaling (BWS) was used to elicit relative preferences across alcohol, tobacco, and cannabis forms. Product-level indices for liking, addictiveness, and risk were analyzed using fractional logistic regression.

**Results:**

Cannabis edibles, alcohol, and cannabis flower ranked highest in liking, while tobacco products—especially cigarettes—were less preferred. E-cigarettes and oral nicotine pouches were more favored than cigarettes but remained less liked than cannabis products. Among cannabis forms, cartridges were perceived as both the most addictive and risky, whereas topicals were viewed as least harmful. Regression results showed cannabis oil and most tobacco products were less liked than flowers, while topicals were perceived as less addictive and risky than flowers.

**Conclusions:**

AYAs favor cannabis edibles and alcohol while disfavoring traditional tobacco products. Perceptions of risk and addictiveness align with product delivery modes, particularly for high-THC vaping products. These findings suggest both substitution (e.g., away from cigarettes) and complementarity (e.g., cannabis and alcohol), underscoring the need for integrated, multi-product regulatory strategies targeting evolving substance markets.

## Introduction

Substance use among U.S. adolescents and young adults (AYAs) remains a central public health concern (National Institutes of Health, 2023, 2024). In 2023, the prevalence of drinking alcohol, using cannabis, and using tobacco products among U.S. high school students in the past 30 days was 22%, 17%, and 13%, respectively (Birdsey et al., 2023; CDC, 2025). Multiple substances are often used together by AYAs, with cannabis surpassing tobacco in the use prevalence and popularity. At the same time, alcohol continues to be one of the most commonly used substances among AYAs and frequently co-occurs with cannabis and nicotine use, raising concerns about multiple substance use and related harms (Terry-McElrath & Patrick, 2018). While national surveillance shows declining youth tobacco use, especially cigarettes, concerns remain about persistent e-cigarette use and the use of novel oral nicotine pouches (Jamal et al., 2024). These shifts occur alongside a rapidly evolving product marketplace spanning nicotine, alcohol, and a widening array of cannabis formulations (e.g., edibles, cartridges, and concentrates). Currently, cannabis and tobacco share similar delivery modes—both products can be consumed *via* vaping, smoking, or digestion, raising concerns about co-use. Understanding how AYAs evaluate these products, what they like most and least, and which they perceive as most addictive or risky can inform prevention and regulatory strategies calibrated to current use patterns.

Few studies have directly compared AYAs’ evaluations of cannabis, nicotine, and alcohol at the product level within a single analytic framework, leaving little evidence on how AYAs simultaneously prioritize products across categories (Bedillion et al., 2024; Chen-Sankey et al., 2024; Choi et al., 2024; Evans-Polce et al., 2025; Florimbio et al., 2023, 2024; Pulvers et al., 2024). Surveillance and cohort studies have described the rising prevalence of cannabis vaping among young adults and the frequent co-use of alcohol, nicotine, and cannabis, with simultaneous use linked to greater acute and chronic risks (Farrelly et al., 2025; Fleming et al., 2021; Lee et al., 2022; Patrick et al., 2024; Pulvers et al., 2024; Romm et al., 2024). Research has also shown that perceived harm, advertising exposure, and price-based promotions are associated with cannabis use and initiation among AYAs, underscoring the policy salience of perceptions and marketing environments (Harrison et al., 2024; Xu et al., 2026). However, most studies stop short of directly quantifying how AYAs rank competing substances and product formats on “liking,” risk, and perceived addictiveness.

A second gap concerns the product attributes that shape preferences. Discrete choice and best-worst scaling (BWS) studies in adult markets suggest that price, tetrahydrocannabinol (THC) content, labeling, and product type strongly influence cannabis purchasing, and that perceived risk varies by the method of administration (Choi et al., 2024; Donnan et al., 2024; Florimbio et al., 2023; Zhu et al., 2021). However, comparable choice-based evidence focused on the U.S. AYAs, spanning cannabis, nicotine (including newer pouches), and alcohol within a single design are scarce. Moreover, few studies have paired preference elicitations (e.g., discrete choice experiments with BWS, or DCE/BWS) with multivariable modeling of the socioeconomic and behavioral correlates of product-level “most/least liked,” “most/least risky,” or “most/least addictive” selections. Most cannabis DCE/BWS studies on adults focus on attribute utilities (price, THC, labeling, product type) rather than respondent-level covariate profiles (Donnan et al., 2024; Zhu et al., 2021). Recent reviews of youth/young adult literature have noted limited product-resolved preference analyses with covariate modeling (Harrison et al., 2024; Kourgiantakis et al., 2024).

Meanwhile, market conditions and regulatory frameworks are in a period of transition (Cooper & Shi, 2025a; FDA, 2025a; Pacula et al., 2021). THC potency has increased over the decades, and several jurisdictions have implemented or debated potency caps and potency-based taxation to mitigate harm concentrated in high-THC products (ElSohly et al., 2024). Regulators are also considering packaging reforms for edibles to reduce youth appeal, improve warning recall, and temper perceived safety, while tobacco control continues to grapple with ENDS regulation and the growth of oral nicotine pouches (Cooper & Shi, 2025a, 2025b; Strongin et al., 2023). These developments heighten the need for contemporary AYA data on product perception and prioritization.

In this study, we address these gaps by conducting a national survey in the U.S. AYAs (ages 15–21) who have used cannabis in the past month or are susceptible to use, and by applying BWS to elicit relative preferences across alcohol, multiple nicotine products (including e-cigarettes and nicotine pouches), and diverse cannabis forms (flower, edibles, cartridges, concentrates, prerolls, and topicals). We paired BWS indices with fractional logistic regression to profile demographic and behavioral correlates of product “liking,” perceived addictiveness, and perceived risk. By offering a cross-substance, product-resolved view grounded in contemporary AYA perceptions, this study updates the evidence base needed to calibrate consumer-facing policies, pricing, taxation, potency limits, and packaging/warning standards to current market realities.

## Method

### Sample and recruitment

We recruited a nationally representative sample of non-institutionalized U.S. adolescents and young adults (AYAs) aged 15–20 years in June 2024 through the Ipsos Knowledge Panel. Panel members are recruited using random probability sampling methods, primarily address-based sampling of residential households, which allows nearly all U.S. households a known probability of selection. Additional details on panel recruitment, weighting, and survey administration are available in prior work using the same panel framework (Xu et al., 2026). Eligible adolescents were identified through parents or legal guardians who were existing Knowledge Panel members and had an age-eligible adolescent residing in their household. One eligible AYA per household was invited to participate in the survey. Participants aged 18 years or older provided electronic informed consent before participation, whereas parental permission and adolescent assent were obtained electronically for participants younger than 18 years. The study protocol was approved by the Institutional Review Board at The Ohio State University.

AYAs were eligible if they reported cannabis use during the past 30 days or were classified as susceptible to future cannabis use. Respondents reporting past 30-day cannabis use were those who answered “Yes” to the survey question asking whether they had used cannabis during the past 30 days. Susceptibility was measured using an established measure that assessed willingness to use cannabis, intention to use, curiosity, anticipated pleasurable effects, and anticipated negative effects of cannabis use. A participant was classified as susceptible if they did not express strong disagreement with cannabis use or its perceived pleasurable effects in any of these areas (Barrington-Trimis et al., 2020). Participants who neither reported past 30-day cannabis use nor met the susceptibility criteria were excluded from the study. These exclusion rules ensured that the final sample consisted only of individuals who either reported cannabis during the past 30 days or met the established criteria for susceptibility to cannabis use.

We focused on AYAs who currently use cannabis or susceptible to cannabis use because they are the population most likely to encounter emerging cannabis products and engage in future cannabis use. Restricting the sample to respondents who reported past 30-day cannabis use and those susceptible to cannabis use allowed us to evaluate preferences across diverse cannabis products and their relationships with alcohol and nicotine products among individuals at greatest risk of polysubstance use.

The final sample included 1,010 respondents, comprising 498 respondents who had ever used cannabis and 512 respondents who had never used cannabis but met the established criteria for susceptibility to future cannabis use. Among the 498 respondents who had ever used cannabis, 247 reported cannabis use during the past 30 days. For the pooled product-level analyses, the respondent-level dataset was expanded to a respondent-by-product dataset, resulting in 9,090 observations (1,010 respondents × 9 products) for product preference analyses and 6,060 observations (1,010 respondents × 6 products) for analyses of perceived addictiveness and perceived risk. The survey collected detailed information on cannabis consumption and spending behaviors, along with key sociodemographic characteristics (e.g., household income and employment). In addition to cannabis-related measures, the survey also collected information on participants’ evaluations of alcohol, tobacco, and nicotine products. Respondents also reported their perceptions and attitudes toward cannabis and other substances, including measures of social acceptability, perceived addictiveness, and perceived risk of harm.

Respondents completed a series of BWS choice tasks in which they viewed subsets of alcohol, tobacco, nicotine, and cannabis products and identified the ‘best’ and ‘worst’ option according to the study objective (liking, perceived addictiveness, or perceived risk). Respondents evaluated all products included in the BWS tasks regardless of their personal use history, allowing the study to capture relative preferences and perceptions shaped by both direct experience and indirect exposure.

### Outcome variables

#### Relative liking (Best-worst scaling, BWS)

Respondents selected the product they liked the most (“best”) and the product they liked the least (“worst”) from a set of products that included alcohol, nicotine/tobacco products (e.g., cigarettes, e-cigarettes, oral nicotine pouches, other tobacco products), and multiple cannabis forms (e.g., flower, preroll, concentrate, cartridge, edible, topical). For each product, we constructed an individual-level BWS score from the “most like” and “least like” selections. Scores summarize the aggregated frequency with which a product was chosen as “most” versus “least” across the respondent’s tasks, scaled to the unit interval [0, 1] for fractional modeling. Higher BWS scores indicate a stronger relative liking for the product.

#### Perceived addictiveness (BWS)

Perceptions of addictiveness were elicited through a survey question in which respondents identified the cannabis product they considered most addictive and least addictive among diverse cannabis forms (e.g., flower, preroll, concentrate, cartridge, edible, topical). We computed product-specific BWS scores rescaled to [0, 1], with higher values indicating greater perceived addictiveness.

#### Perceived risk (BWS)

Perceived risk was measured using a similar survey question in which respondents selected the cannabis product they considered most risky and least risky among multiple cannabis forms (same as above). Product-level scores were computed in the same manner and rescaled to [0,1], with higher values indicating a greater perceived risk.

### Key predictors

#### Product category

In models that benchmark relative liking or perceptions across products, we included product indicators with cannabis flowers as the reference category to obtain the average marginal effects for each product.

#### Past 30-day cannabis use

Indicator for past 30-day cannabis use (yes/no), used to capture behavioral proximity to cannabis markets and to examine whether current use was related to preference or perception scores.

#### Covariates

We adjusted for a consistent set of demographic and socioeconomic characteristics, including age in years, gender (male, female as reference), race/ethnicity (black, non-Hispanic; other, non-Hispanic; 2+ races, non-Hispanic; Hispanic; White, non-Hispanic as reference), education (high school graduate or equivalent, some college or associate’s degree, bachelor’s degree or above, no high school diploma as reference), employment (working part time, not working, working full time as reference), household income ($50,000 to $99,999, $100,000 to $149,999, $150,000 or more, 0 to $49,999 as reference), and region (Midwest, South, West, Northeast as reference) (U.S. Census Bureau, 2010).

#### BWS construction

To construct the regression outcome, individual responses within each BWS task were first coded using the conventional BWS coding scheme (+1 for “most”, 0 for “not selected”, and −1 for “least”). These coded responses were then aggregated across all BWS choice tasks for each product to obtain a product-specific BWS score for each respondent. Finally, the aggregated scores were linearly rescaled to the unit interval [0,1]. Thus, the dependent variable used in the regression is a continuous bounded score rather than the original three-category BWS response. This transformation preserves the relative ordering and information contained in the original BWS responses, making the outcome suitable for fractional logistic regression models designed for proportional or bounded dependent variables.

For the BWS items, responses of “Don’t know” and “Refused” were treated as missing, because they do not represent valid best-worst selections. Respondents who considered all products equally liked, addictive, or risky selected the corresponding “all products are equally…” option, which was treated as a valid substantive response. Therefore, coding “Don’t know” or “Refused” as neutral (midpoint) responses would incorrectly equate lack of knowledge with equal evaluation. Table A8 compares respondents included in the analytical sample with those whose BWS responses were coded as missing. No statistically significant differences were observed across the examined sociodemographic characteristics. This approach to constructing BWS scores is consistent with established BWS methodologies used in health economics and behavioral research (Flynn et al., 2007; Louviere et al., 2015).

#### Regression analysis

We modeled the association between respondent characteristics and the aggregated product-specific BWS scores using fractional logistic regression, which are appropriate for dependent variables bounded in the unit interval [0, 1], including normalized indices derived from survey-based measures (Mullahy, 2015; Papke & Wooldridge, 2008). The three dependent variables were the best–worst scores for (i) relative liking, (ii) perceived addictiveness, and (iii) perceived risk (each rescaled to the unit interval, as described in the BWS construction).

Our primary pooled specification leveraged the respondent–by–product panel and included product indicators (to compare products relative to a reference category) and respondent fixed effects (to absorb unobserved, time-invariant heterogeneity in response style and baseline preferences). Formally, the conditional mean of the BWS outcome for individual *i* and product *p* was modeled *via* a fractional logistic regression as a function of product dummies and respondent effects.


$$logit\;\left(u_{ip}\right)=\alpha_{i}+D_{p}^{'}\gamma +X_{i}^{\prime }\beta$$

In this fractional logistic regression, $u_{ip}$ denotes the conditional mean of BWS index for individual i and product $p,\;D_{p}$ is the vector of product indicators, and $\alpha_{i}$ is the respondent-specific fixed effect. In addition, we conducted separate regressions for each product or form, where individual fixed effects are replaced by $X_{i}$, the vector of observed covariates (demographic, socioeconomic for individual i.

We report average marginal effects (AMEs) from fractional logistic regressions, with standard errors in parentheses clustered at the individual level. In the pooled models, AMEs quantify the average difference in BWS scores across product categories relative to the reference product, conditional on respondent fixed effects. In the product-specific models, AMEs represent adjusted associations between respondent characteristics and the probability of assigning higher BWS scores to a given product, after controlling for demographic, socioeconomic, and behavioral covariates. All regression models incorporated the survey weights to produce estimates representative of the U.S. non-institutionalized AYA population aged 15–20 years surveyed in June 2024.

## Results

Table 1 presents average BWS scores for perceived likability, addictiveness, and risk, ordered by rank to highlight relative positions across categories. Cannabis edibles are the most likable with a mean likability score (0.422), followed by alcohol (0.403), cannabis flowers (0.396), cannabis vaping (0.371), cannabis-other (0.362), e-cigarettes (0.346), oral nicotine pouches (0.339), tobacco-other (0.294), and cigarettes (0.280). The comparisons of 95% Confidence Intervals (CI) show that alcohol is similarly likeable compared to various cannabis forms. Interestingly, while cigarettes and tobacco-other are significantly less likable than all cannabis forms, newer tobacco products such as e-cigarettes and oral nicotine pouches are similarly likeable as cannabis vaping.

For perceived addictiveness and risks, the ranking of different cannabis forms is the same. Cannabis cartridges for vaping are rated the “most addictive” (0.741) and the “most risky” (0.734), followed by concentrates (addictive score = 0.677, risk score = 0.647), prerolls (addictive score = 0.668, risk score = 0.625), flowers (addictive score = 0.659, risk score = 0.587), edibles (addictive score = 0.640, risk score = 0.537), and topicals (addictive score = 0.512, risk score = 0.463). Based on the 95% CI, cannabis vaping cartridges were statistically rated more addictive and riskier than other forms. Cannabis topicals were rated less addictive and riskier than other forms. In addition, cannabis edibles and flowers were rated less risky than cartridges, concentrates, and prerolls. Tables A1 and A2 further illustrate these patterns, which provide the raw distributions of cannabis products most and least often perceived as likable, addictive, or risky, complementing the mean scores in Table 1. The frequencies of “Don’t know or Refused” responses are reported in Tables A1 and A2. Table A3 presents summary statistics for the BWS scores, and Table A4 details the summary statistics of sociodemographic characteristics.

Table 2 reports the average marginal effects (AME) from fractional logistic regressions that analyzed how the likeable, addictive, and scores are associated with products and forms, using cannabis flower as the reference. Cannabis edibles and alcohol did not statistically differ from cannabis flowers. Cannabis forms that were less liked than flowers include cannabis oil for vaping (AME = −0.137 or 13.7 percentage point less likeable, 95% CI = [−0.208, −0.067], *p* < 0.001) and other cannabis forms (AME = −0.154, 95% CI = [−0.212, −0.095], *p* < 0.001). All nicotine or tobacco products were markedly less likable than flowers, including cigarettes (AME = −0.448, 95% CI = [−0.538, −0.358], *p* < 0.001), tobacco-other, (AME = −0.441, 95% CI = [−0.528, −0.353], *p* < 0.001), oral nicotine pouches (AME = −0.226, 95% CI = [−0.294, −0.158], *p* < 0.001), and e-cigarettes (AME = −0.200, 95% CI = [−0.274, −0.126], *p* < 0.001).

In terms of addictiveness compared to cannabis flowers, concentrates are considered to be similar with no significant differences in addictiveness; cartridge, edibles, and prerolls were considered to be 17.4 (AME = 0.174, 95% CI = [0.130, 0.218], *p* < 0.001), 6.2 (AME = 0.062, 95% CI = [0.023, 0.101], *p* < 0.01), and 4.5 (AME = 0.045, 95% CI = [0.010, 0.081], *p* < 0.05) percentage point more addictive, respectively; and topicals were perceived as 32.7 percentage point less addictive (AME = −0.327, 95% CI = [−0.394, −0.260], *p* < 0.001). When comparing the perceived risks of other cannabis forms with that of flowers, cartridges, edible, prerolls, and concentrates were considered 35.7 percentage point more risky (AME = 0.357, 95% CI = [0.301, 0.413], *p* < 0.001), 20.9 percentage point more risky (AME = 0.209, 95% CI = [0.160, 0.258], *p* < 0.001), 17.6 percentage point more risky (AME = 0.176, 95% CI = [0.125, 0.227], *p* < 0.001), and 9.7 percentage point more risky (AME = 0.091, 95% CI = [0.025, 0.157], *p* < 0.01), respectively. In contrast, topicals were perceived as 19.2 percentage point less risky than flowers (AME = −0.192, 95% CI = [−0.269, −0.115]). To complement the main analyses, Tables A5–A7 present categorical BWS models adjusted for sociodemographic and other factors to the likelihood that each product is selected as most liked, most addictive, or most risky. In Table A5, past-30-day cannabis use is a strong correlate of product liking (e.g., favoring edible and flower), with a few demographic differences (e.g., higher income linked to perceiving concentrates as riskier compared to respondents with income less than $50,000). Moreover, we observed lower alcohol liking among those living in Midwest (AME = −0.370, *p* < 0.05), South (AME = −0.428, *p* < 0.01), or West (AME = −0.632, *p* < 0.001), than respondents living in the Northeast. Race/Ethnicity also serves as an important factor.

Table A6 shows that demographic patterns are generally not statistically significant, with a few exceptions. Respondents with 2+ races non-Hispanic were less likely to rate cannabis cartridge (AME = −0.538, *p* < 0.01) and preroll (AME = −0.442, *p* < 0.01) as “most addictive” than White non-Hispanic. Table A7 highlights the perceived risk patterns. Black, non-Hispanic respondents more often rated flowers as most risky (AME = 0.375, *p* < 0.05) than White, non-Hispanic. Men were more likely to choose topicals as the riskiest than women (AME = 0.299, *p* < 0.05). Higher household income was linked to perceiving concentrates as riskiest ($100,000 to $149,999: AME = 0.450, *p* < 0.05) than respondents with household income less than $50,000.

## Discussion

AYAs are at higher risks of addiction compared to adults and are known to experiment with multiple substances (Chambers et al., 2003; Squeglia & Cservenka, 2017). In recent years, U.S. surveillance shows historically low youth cigarette smoking, but the alcohol drinking prevalence remains high. Meanwhile, the prevalence of cannabis use has risen and reached historical high due to increasing legalization (CDC, 2024; FDA, 2025b). These contrasting trends call for a paradigm shift when investigating multiple substance use among AYAs, which has been predominantly focusing on AYAs who are susceptible to tobacco or alcohol use or whether these products are gateway products for cannabis. To address this gap, this study recruits a nationally representative sample of AYAs who are using or susceptible to use cannabis and examine their product preference among a series of substances and different cannabis forms. The benchmarking using cannabis use and susceptibility captures the shifts in popularity from tobacco to cannabis, which will shed light on regulations over these substances.

We found that cannabis edibles, alcohol, and cannabis flowers were the most likable products for AYAs, in line with the high prevalence reported in national surveys (Evans-Polce et al., 2024; Sun et al., 2022). The finding suggests the continued central role of alcohol in AYA substance use patterns, even when the tobacco and cannabis marketplaces have undergone significant changes in terms of product varieties and availability (Spillane et al., 2025; Spindle et al., 2019). The finding is also in line with the evidence that cannabis and alcohol products are likely complements. We also found that cigarettes and oral nicotine pouches are significantly more likeable than cigarettes and other tobacco, a pattern in line with the relative popularity and use prevalence trends among AYAs (Lee et al., 2022; Waddell et al., 2023; Zhu et al., 2025). Specifically, while the prevalence of oral nicotine pouches has been increasing among AYAs, and e-cigarettes have replaced cigarettes to be the most popular product in this population.

In terms of perceived addictiveness and risks among cannabis forms, cartridges were most consistently identified as the product with the most risky and addictive, followed by concentrates, prerolls, flowers, and edibles, whereas topicals were viewed as substantially less addictive and risky. These perceptions generally align with THC levels, delivery modes, and the psychoactive impacts of these products, as inhaled and high-THC forms deliver rapid cannabinoid absorption and often at higher THC levels (Poyatos et al., 2020). It is also likely that the Evali outbreak associated with THC vaping and the widespread of nicotine vaping (i.e., e-cigarettes) addiction and risks may contribute to the perceptions of cannabis vaping.

Although cannabis flowers and edibles generally contain lower THC concentrations than concentrates or cartridges, they are not necessarily safer (Johnston et al., 2022). Flowers are often smoked that could lead to exposure to carcinogens and other harmful chemicals and result in adverse respiratory health effects. Edibles are linked to overdose and accidental ingestion among children. Perceptions of relative likability, addictiveness, and risk of these forms can foster earlier experimentation and broader uptake, particularly as cannabis becomes increasingly legal in the US. Consequently, regulatory frameworks may consider warning and messages that inform the risks of various cannabis forms to prevent AYA use.

Furthermore, neuroscience research suggests that AYAs are biologically more susceptible to addiction due to heightened reward sensitivity, which may contribute to the multiple substance use among this age group (Chambers et al., 2003; Spear, 2018; Squeglia & Cservenka, 2017; Volkow & Boyle, 2018). Our findings further demonstrate that AYAs’ assessments on relative appeals, risks, and addictions among substances show statistically significant rankings. Regulatory policies should therefore extend beyond single products to account for patterns of complementarity and substitution across substances with other substances. Regulations that narrowly target one product risk shifting consumption to alternative forms or to other substances, reinforcing the importance of coordinated multi-product strategies.

This study had several limitations. The analysis was cross-sectional (wave 1) and relied on self-reported behaviors and perceptions. BWS tasks capture relative valuations instead of absolute variations, and because respondents evaluated all products regardless of prior use, some responses may have reflected perceptions formed through indirect exposure (e.g., peers, family, or media) rather than personal experience. Nevertheless, these perceptions remain relevant because they may influence future experimentation and product choice among AYAs. The sample was restricted to AYAs using or susceptible to cannabis, which may limit generalizability. Additionally, because the survey was conducted at a single point in time and within a specific policy environment, the findings may not reflect changes in perceptions and behaviors as cannabis markets and regulations continue to evolve.

Despite these limitations, this study advances the evidence base by providing a scalable framework that integrates BWS with regression modeling of sociodemographic and behavioral correlates across cannabis, nicotine, and alcohol products. Our findings highlight how product format and delivery mode shape youth perceptions and preferences across substances. From a policy perspective, the popularity of edibles and the perceived risks of high-THC vaping products underscore the need for targeted prevention strategies, including clearer risk communication, packaging regulations, and monitoring of emerging product forms. Next steps include following the cohort longitudinally, linking to jurisdictional policy and retail data (price, potency, packaging), and testing how changes in regulation shift cross-product liking and perceived risk/addictiveness, particularly for edibles and high-THC vape/concentrate products flagged in this study.

## Figures and Tables

**Table 1.** Summary statistics for best-worst scaling scores (higer scores indicate a product was selected more frequently as “most” and less frequently as “least” across tasks), *N* = 1,010.

Rank	Product	Likable score	Product	Addictive score	Risky score
		*Mean [95% CI]*		*Mean [95% CI]*	*Mean [95% CI]*

1	Cannabis edible	0.422 [0.401, 0.442]	Cannabis cartridge	0.741 [0.725, 0.757]	0.734 [0.718, 0.750]
2	Alcohol	0.403 [0.383, 0.423]	Cannabis concentrate	0.677 [0.661, 0.693]	0.647 [0.632, 0.662]
3	Cannabis flowers	0.396 [0.375, 0.417]	Cannabis Preroll	0.668 [0.653, 0.683]	0.625 [0.611, 0.639]
4	Cannabis oil (vaping)	0.371 [0.353, 0.388]	Cannabis flower	0.659 [0.642, 0.677]	0.587 [0.568, 0.606]
5	Cannabis other	0.362 [0.347, 0.378]	Cannabis edible	0.640 [0.622, 0.658]	0.537 [0.517, 0.556]
6	E-cigarettes	0.346 [0.328, 0.363]	Cannabis topicals	0.512 [0.489, 0.536]	0.463 [0.442, 0.485]
7	Oral nicotine pouches	0.339 [0.324, 0.355]			
8	Tobacco (other)	0.294 [0.277, 0.310]			
9	Cigarettes	0.280 [0.264, 0.297]			

*Note:* CI represents confidence interval.

**Table 2.** Average marginal effects of product category on best-Worst outcomes (likable, addictive, and risky scores).

	Average marginal effect		
	(1)	(2)	(3)

Categories (base group: cannabis flower)	Likable	Addictive	Risky
Cannabis cartridge		0.174*** (0.023)	0.357*** (0.028)
Cannabis edible	0.057 (0.044)	0.062** (0.020)	0.209*** (0.025)
Cannabis concentrate		0.028 (0.024)	0.091** (0.034)
Cannabis prerolls		0.045* (0.018)	0.176*** (0.026)
Cannabis topicals		−0.327*** (0.034)	−0.192*** (0.040)
Other categories (likable only)			
Alcohol	−0.019 (0.041)		
Cannabis oil	−0.137*** (0.036)		
Cannabis other	−0.154*** (0.030)		
Cigarettes	−0.448*** (0.046)		
E-cigarettes	−0.200*** (0.038)		
Oral nicotine pouches	−0.226*** (0.035)		
Tobacco (other)	−0.441*** (0.045)		
Observations	9090	6060	6060
Case ID	1010	1010	1010

*Notes*: Product categories are different between model (1) and model (2)-(3). Models (2) and (3) include cannabis product forms only because perceived addictiveness and risk were assessed for cannabis products only. Standard errors are clustered at the individual level and shown in parentheses.

* *p* < 0.05,

** *p* < 0.01,

*** *p* < 0.001.

## Appendix A

**Table A1.** Distribution of most and least liked products (*N* = 1,010).

	Most liked	Least liked

Alcohol	113 (11.19%)	19 (1.88%)
Cannabis edible	146 (14.46%)	14 (1.39%)
Cannabis flower	129 (12.77%)	49 (4.85%)
Cannabis oil	55 (5.45%)	26 (2.57%)
Cannabis other	15 (1.49%)	3 (0.30%)
Cigarettes	6 (0.59%)	160 (15.84%)
E-Cigarettes	33 (3.27%)	55 (5.45%)
Like equal	14 (1.39%)	14 (1.39%)
Dislike equal	304 (30.10%)	304 (30.10%)
Oral nicotine pouches	3 (0.30%)	38 (3.76%)
Nicotine (other)	2 (0.20%)	129 (12.77%)
Don’t know or refused	190 (18.81%)	199 (19.70%)
Total	1010	1010

**Table A2.** Distribution of most and least addictive and risky cannabis products (*N* = 1,010).

	Most addictive	Least addictive	Most risky	Least ricky

Cannabis edible	52 (5.15%)	68 (6.73%)	47 (4.65%)	170 (16.83%)
Cannabis flower	79 (7.82%)	55 (5.45%)	98 (9.70%)	120 (11.88%)
Cannabis concentrate	76 (7.52%)	16 (1.58%)	113 (11.19%)	14 (1.39%)
Cannabis cartridge	196 (19.41%)	7 (0.69%)	286 (28.32%)	10 (0.99%)
Cannabis preroll	51 (5.05%)	9 (0.89%)	65 (6.44%)	9 (0.89%)
Cannabis topical	7 (0.69%)	280 (27.72%)	8 (0.79%)	279 (27.62%)
Equal addictive	298 (29.50%)	298 (29.50%)	197 (19.50%)	197 (19.50%)
Don’t know or refused	251 (24.85%)	277 (27.43%)	196 (19.41%)	211 (20.89%)
Total	1010	1010	1010	1010

**Table A3.** Distribution of task-level BWS coding for substance preferences and perceived risk and addictiveness (*N* = 1,010).

		Score frequency (%)		
		Least (0)	Neutral (0.5)	Most (1)

“Most Liked” product preferences	Alcohol	323 (31.98%)	560 (55.45%)	127 (12.57%)
	Cannabis edible	318 (31.49%)	532 (52.67%)	160 (15.84%)
	Cannabis flower	353 (34.95%)	514 (50.89%)	143 (14.16%)
	Cannabis oil	330 (32.67%)	611 (60.50%)	69 (6.83%)
	Cannabis other	307 (30.40%)	674 (66.73%)	29 (2.87%)
	Cigarettes	464 (45.94%)	526 (52.08%)	20 (1.98%)
	E-Cigarettes	359 (35.54%)	604 (59.80%)	47 (4.65%)
	Oral nicotine pouches	342 (33.86%)	651 (64.46%)	17 (1.68%)
	Tobacco (other)	433 (42.87%)	561 (55.54%)	16 (1.58%)
Addictive perception product preferences	Cannabis edible	68 (6.73%)	592 (58.61%)	350 (34.65%)
	Cannabis flower	55 (5.45%)	578 (57.23%)	377 (37.33%)
	Cannabis concentrate	16 (1.58%)	620 (61.39%)	374 (37.03%)
	Cannabis cartridge	7 (0.69%)	509 (50.40%)	494 (48.91%)
	Cannabis preroll	9 (0.89%)	652 (64.55%)	349 (34.55%)
	Cannabis topical	280 (27.72%)	425 (42.08%)	305 (30.20%)
Risky perception product preferences	Cannabis edible	170 (16.83%)	596 (59.01%)	244 (24.16%)
	Cannabis flower	120 (11.88%)	595 (58.91%)	295 (29.21%)
	Cannabis concentrate	14 (1.39%)	686 (67.92%)	310 (30.69%)
	Cannabis cartridge	10 (0.99%)	517 (51.19%)	483 (47.82%)
	Cannabis preroll	9 (0.89%)	739 (73.17%)	262 (25.94%)
	Cannabis topical	279 (27.62%)	526 (52.08%)	205 (20.30%)

*Notes*: These values represent the coding of individual BWS task responses before aggregation. The regression analyses use aggregated product-specific BWS scores that are continuous and bounded between 0 and 1.

**Table A4.** Summary statistics of sociodemographic characteristics (*N* = 1,010).

Variables	Mean (SD)/Percent

Age	17.68 (1.69)
Household size	4.02 (1.48)
Gender	
Female	54.62%
Male	45.38%
Race/Ethnicity	
White, non-Hispanic	59.21%
Black, non-Hispanic	11.39%
Other, non-Hispanic	3.07%
Hispanic	18.61%
2+ Races, non-Hispanic	7.72%
Education	
No high school diploma or GED	43.27%
High school graduate (high school diploma or the equivalent GED)	28.22%
Some college or Associate’s degree	25.45%
Bachelor’s degree or above	3.07%
Household income	
Less than $50,000	24.26%
$50,000 to $99,999	29.10%
$100,000 to $149,999	21.39%
$150,000 or more	25.25%
Region	
Northeast	16.24%
Midwest	23.76%
South	38.02%
West	21.98%
Employment	
Working full-time	16.85%
Working part-time	40.96%
Not working	42.19%
Cannabis use	
Current use (past 30-day use)	24.46%
Former use (no past 30-day use)	24.85%
Never use (Susceptible group)	50.69%

**Table A5.** Logistic regression of best-worst scaling scores for “most liked” product preferences by demographic and socioeconomic characteristics (part I, II, and III).

	(1)		(2)		(3)	
	Alcohol		Cannabis edible		Cannabis flowers	

**Age**	0.003	(0.047)	−0.019	(0.062)	0.071	(0.072)
**Gender (ref: Female)**						
Male	−0.157	(0.119)	−0.122	(0.143)	−0.082	(0.167)
**Race/Ethnicity (ref: White, Non-Hispanic)**						
Black, non-Hispanic	0.029	(0.183)	−0.229	(0.174)	0.265	(0.289)
Other, non-Hispanic	−0.582	(0.355)	−0.751	(0.474)	−1.059	(0.570)
Hispanic	0.260	(0.155)	0.385	(0.198)	0.126	(0.202)
2+ Races, non-Hispanic	0.039	(0.232)	−0.011	(0.297)	0.221	(0.323)
**Household size**	−0.012	(0.037)	−0.007	(0.040)	0.027	(0.047)
**Education (ref: No high school diploma or GED)**						
High school graduate (high school diploma or the equivalent GED)	−0.112	(0.159)	0.074	(0.206)	−0.257	(0.248)
Some college or Associate’s degree	0.362*	(0.181)	0.028	(0.227)	−0.231	(0.283)
Bachelor’s degree or above	0.551	(0.361)	0.174	(0.345)	−0.298	(0.414)
**Household income (ref: 0 to $49,999)**						
$50,000 to $99,999	0.193	(0.147)	0.277	(0.192)	0.081	(0.222)
$100,000 to $149,999	−0.005	(0.210)	0.059	(0.201)	−0.292	(0.260)
$150,000 or more	0.318	(0.163)	0.336	(0.191)	−0.300	(0.244)
**Employment (ref: Working full-time)**						
Working part-time	−0.131	(0.187)	0.138	(0.190)	−0.159	(0.226)
Not working	−0.119	(0.201)	0.302	(0.204)	0.040	(0.264)
**Region (ref: Northeast)**						
Midwest	−0.370*	(0.168)	−0.171	(0.183)	0.142	(0.235)
South	−0.428**	(0.148)	−0.272	(0.167)	−0.265	(0.198)
West	−0.632***	(0.180)	−0.534**	(0.187)	−0.460	(0.243)
**Past 30-day cannabis use (ref: No)**						
Yes	0.156	(0.118)	0.393**	(0.137)	0.990***	(0.162)
Observations	486		486		486	

**Table A6.** Fractional logistic regression of best-worst scaling scores for “most addictive” product preferences by demographic and socioeconomic characteristics (part I and II).

	(1)		(2)		(3)	
	Cannabis cartridge		Cannabis edible		Cannabis flowers	

**Age**	−0.009	(0.055)	−0.059	(0.048)	−0.038	(0.051)
**Gender (ref: Female)**	0.052	(0.129)	0.009	(0.115)	0.037	(0.122)
Male						
**Race/Ethnicity (ref: White, Non-Hispanic)**						
Black, non-Hispanic	−0.290	(0.202)	0.026	(0.179)	−0.079	(0.191)
Other, non-Hispanic	0.119	(0.451)	−0.097	(0.414)	0.624	(0.415)
Hispanic	0.006	(0.175)	−0.076	(0.159)	0.066	(0.163)
2+ Races, non-Hispanic	−0.538**	(0.196)	−0.297	(0.188)	−0.260	(0.185)
**Household Size**	−0.011	(0.040)	−0.016	(0.036)	−0.002	(0.041)
**Education (ref: No high school diploma or GED)**						
High school graduate (high school diploma or the equivalent GED)	0.303	(0.203)	0.177	(0.189)	0.064	(0.189)
Some college or Associate’s degree	0.076	(0.214)	0.050	(0.189)	−0.151	(0.214)
Bachelor’s degree or above	0.275	(0.371)	−0.179	(0.299)	0.014	(0.319)
**Household income (ref: 0 to $49,999)**						
$50,000 to $99,999	0.046	(0.167)	−0.299	(0.154)	−0.022	(0.157)
$100,000 to $149,999	0.012	(0.201)	−0.300	(0.182)	−0.107	(0.184)
$150,000 or more	0.013	(0.181)	−0.270	(0.160)	−0.168	(0.172)
**Employment (ref: Working full-time)**						
Working part-time	0.302	(0.165)	0.152	(0.139)	0.056	(0.155)
Not working	0.034	(0.168)	0.050	(0.151)	−0.076	(0.165)
**Region (ref: Northeast)**						
Midwest	−0.294	(0.227)	−0.069	(0.193)	−0.065	(0.200)
South	−0.385	(0.198)	−0.257	(0.166)	−0.251	(0.174)
West	−0.078	(0.224)	0.122	(0.201)	−0.082	(0.201)
**Past 30-day cannabis use (ref: No)**						
Yes	−0.007	(0.127)	0.011	(0.111)	−0.010	(0.118)
Observations	486		486		486	

**Table A7.** Fractional logistic regression of best-worst scaling scores for “most risky” product Preferences by demographic and socioeconomic characteristics (part I and II).

	(1)		(2)		(3)	
	Cannabis cartridge		Cannabis edible		Cannabis flowers	

**Age**	0.048	(0.053)	−0.028	(0.042)	0.040	(0.053)
**Gender (ref: Female)**						
Male	0.131	(0.129)	0.061	(0.099)	−0.011	(0.121)
**Race/Ethnicity (ref: White, Non-Hispanic)**						
Black, non-Hispanic	−0.074	(0.231)	0.020	(0.183)	0.375*	(0.184)
Other, non-Hispanic	0.043	(0.357)	0.124	(0.321)	−0.085	(0.365)
Hispanic	0.106	(0.173)	−0.050	(0.129)	0.115	(0.158)
2+ Races, non-Hispanic	−0.060	(0.228)	−0.144	(0.161)	0.072	(0.220)
**Household Size**	−0.013	(0.040)	−0.008	(0.031)	−0.011	(0.036)
**Education (ref: No high school diploma or GED)**						
High school graduate (high school diploma or the equivalent GED)	−0.022	(0.190)	0.222	(0.157)	−0.020	(0.201)
Some college or Associate’s degree	0.019	(0.211)	0.060	(0.167)	−0.199	(0.206)
Bachelor’s degree or above	−0.270	(0.338)	−0.195	(0.279)	−0.380	(0.302)
**Household income (ref: 0 to $49,999)**						
$50,000 to $99,999	0.036	(0.168)	0.118	(0.136)	0.167	(0.152)
$100,000 to $149,999	−0.147	(0.188)	0.275	(0.168)	0.050	(0.191)
$150,000 or more	−0.076	(0.180)	−0.024	(0.130)	−0.141	(0.162)
**Employment (ref: Working full-time)**						
Working part-time	0.003	(0.165)	0.064	(0.125)	−0.230	(0.148)
Not working	−0.014	(0.172)	0.137	(0.141)	−0.253	(0.158)
**Region (ref: Northeast)**						
Midwest	−0.033	(0.212)	0.191	(0.171)	0.069	(0.194)
South	−0.130	(0.189)	0.087	(0.151)	0.019	(0.177)
West	−0.098	(0.211)	0.140	(0.165)	−0.063	(0.199)
**Past 30-day cannabis use (ref: No)**						
Yes	−0.292*	(0.127)	0.132	(0.096)	−0.215	(0.117)
Observations	486		486		486	

	(1)		(2)		(3)	
	Cannabis oil		Cannabis other		Cigarettes	

**Age**	−0.066	(0.057)	−0.019	(0.040)	0.052	(0.056)
**Gender (ref: Female)**						
Male	−0.191	(0.126)	−0.155	(0.099)	−0.191	(0.131)
**Race/Ethnicity (ref: White, Non-Hispanic)**						
Black, non-Hispanic	−0.102	(0.155)	0.045	(0.145)	−0.208	(0.209)
Other, non-Hispanic	−0.243	(0.571)	−0.464	(0.400)	−0.078	(0.418)
Hispanic	0.183	(0.176)	0.183	(0.122)	0.445**	(0.170)
2+ Races, non-Hispanic	−0.134	(0.188)	−0.075	(0.170)	−0.508	(0.321)
**Household Size**	0.045	(0.041)	−0.017	(0.028)	−0.036	(0.040)
**Education (ref: No high school diploma or GED)**						
High school graduate (high school diploma or the equivalent GED)	0.224	(0.193)	−0.006	(0.136)	−0.298	(0.208)
Some college or Associate’s degree	0.267	(0.214)	0.125	(0.154)	−0.239	(0.221)
Bachelor’s degree or above	0.082	(0.259)	0.312	(0.306)	−0.285	(0.328)
**Household income (ref: 0 to $49,999)**						
$50,000 to $99,999	0.132	(0.166)	0.197	(0.135)	0.168	(0.184)
$100,000 to $149,999	−0.064	(0.192)	−0.095	(0.170)	−0.101	(0.207)
$150,000 or more	0.129	(0.159)	0.049	(0.127)	0.051	(0.164)
**Employment (ref: Working full-time)**						
Working part-time	−0.012	(0.178)	0.037	(0.152)	−0.113	(0.193)
Not working	0.172	(0.192)	0.110	(0.170)	−0.001	(0.203)
**Region (ref: Northeast)**						
Midwest	0.151	(0.180)	−0.202	(0.127)	−0.256	(0.185)
South	−0.151	(0.152)	−0.310**	(0.112)	−0.118	(0.146)
West	−0.147	(0.183)	−0.457***	(0.135)	−0.663***	(0.180)
**Past 30-day cannabis use (ref: No)**						
Yes	0.541***	(0.126)	0.514***	(0.095)	0.592***	(0.123)
Observations	486		486		486	

	(1)		(2)		(3)	
			
	E-cigarettes		Oral nicotine pouches		Tobacco (other)	

**Age**	0.036	(0.051)	−0.106	(0.066)	0.001	(0.046)
**Gender (ref: Female)**						
Male	−0.240	(0.132)	−0.260	(0.145)	0.007	(0.112)
**Race/Ethnicity (ref: White, Non-Hispanic)**						
Black, non-Hispanic	−0.067	(0.187)	0.295	(0.235)	0.025	(0.182)
Other, non-Hispanic	−0.993*	(0.486)	−0.215	(0.405)	−0.498	(0.402)
Hispanic	0.017	(0.179)	0.284	(0.187)	0.286*	(0.134)
2+ Races, non-Hispanic	−0.279	(0.235)	0.265	(0.211)	0.107	(0.170)
**Household Size**	0.009	(0.033)	−0.093*	(0.044)	−0.040	(0.032)
**Education (ref: No high school diploma or GED)**						
High school graduate (high school diploma or the equivalent GED)	−0.066	(0.184)	0.310	(0.219)	−0.035	(0.158)
Some college or Associate’s degree	−0.079	(0.194)	0.214	(0.253)	0.092	(0.175)
Bachelor’s degree or above	−0.432	(0.338)	0.451	(0.320)	−0.011	(0.298)
**Household income (ref: 0 to $49,999)**						
$50,000 to $99,999	0.078	(0.180)	0.080	(0.198)	−0.028	(0.165)
$100,000 to $149,999	0.034	(0.239)	−0.183	(0.221)	−0.129	(0.179)
$150,000 or more	−0.129	(0.163)	0.076	(0.206)	0.005	(0.135)
**Employment (ref: Working full-time)**						
Working part-time	−0.020	(0.175)	0.138	(0.202)	0.019	(0.168)
Not working	0.167	(0.195)	0.183	(0.223)	0.154	(0.190)
**Region (ref: Northeast)**						
Midwest	0.044	(0.188)	−0.143	(0.201)	−0.141	(0.131)
South	−0.002	(0.182)	−0.307	(0.176)	−0.332**	(0.110)
West	−0.057	(0.227)	−0.415*	(0.208)	−0.451**	(0.140)
**Past 30-day cannabis use (ref: No)**						
Yes	0.254	(0.133)	0.466***	(0.139)	0.326**	(0.102)
Observations	486		486		486	

	(1)		(2)		(3)	
	Cannabis concentrate		Cannabis prerolls		Cannabis topicals	

**Age**	0.038	(0.055)	−0.021	(0.044)	−0.030	(0.061)
**Gender (ref: Female)**						
Male	−0.107	(0.128)	0.088	(0.104)	0.261	(0.148)
**Race/Ethnicity (ref: White, Non-Hispanic)**						
Black, non-Hispanic	−0.005	(0.216)	−0.201	(0.155)	−0.060	(0.230)
Other, non-Hispanic	0.075	(0.366)	−0.046	(0.329)	−0.058	(0.467)
Hispanic	−0.095	(0.186)	−0.174	(0.145)	0.083	(0.199)
2+ Races, non-Hispanic	−0.228	(0.218)	−0.442**	(0.147)	−0.286	(0.256)
Household size	0.038	(0.040)	0.031	(0.033)	−0.012	(0.046)
**Education (ref: No high school diploma or GED)**						
High school graduate (high school diploma or the equivalent GED)	−0.090	(0.203)	0.080	(0.164)	0.207	(0.232)
Some college or Associate’s degree	−0.170	(0.219)	−0.081	(0.172)	−0.203	(0.250)
Bachelor’s degree or above	−0.144	(0.326)	−0.240	(0.243)	−0.218	(0.428)
**Household income (ref: 0 to $49,999)**						
$50,000 to $99,999	−0.017	(0.164)	−0.131	(0.140)	−0.297	(0.192)
$100,000 to $149,999	−0.246	(0.204)	−0.240	(0.156)	−0.400	(0.239)
$150,000 or more	0.058	(0.177)	−0.088	(0.148)	−0.376	(0.209)
**Employment (ref: Working full-time)**						
Working part-time	0.296	(0.163)	0.099	(0.127)	−0.090	(0.184)
Not working	0.221	(0.170)	0.011	(0.135)	−0.175	(0.198)
**Region (ref: Northeast)**						
Midwest	0.013	(0.206)	−0.121	(0.182)	−0.193	(0.254)
South	−0.275	(0.185)	−0.284	(0.159)	−0.296	(0.219)
West	0.042	(0.216)	−0.019	(0.183)	−0.108	(0.248)
**Past 30-day cannabis use (ref: No)**						
Yes	−0.121	(0.122)	−0.163	(0.101)	0.085	(0.146)
Observations	486		486		486	

	(1)		(2)		(3)	
	Cannabis concentrate		Cannabis prerolls		Cannabis topicals	

**Age**	−0.063	(0.055)	−0.009	(0.039)	−0.029	(0.058)
**Gender (ref: Female)**						
Male	0.005	(0.127)	0.055	(0.091)	0.299*	(0.130)
**Race/Ethnicity (ref: White, Non-Hispanic)**						
Black, non-Hispanic	−0.061	(0.213)	0.224	(0.152)	0.303	(0.206)
Other, non-Hispanic	−0.240	(0.334)	−0.181	(0.220)	0.085	(0.420)
Hispanic	−0.108	(0.178)	0.048	(0.124)	0.136	(0.177)
2+ Races, non-Hispanic	−0.010	(0.245)	0.033	(0.153)	0.048	(0.239)
**Household Size**	0.014	(0.039)	−0.008	(0.025)	−0.049	(0.041)
**Education (ref: No high school diploma or GED)**						
High school graduate (high school diploma or the equivalent GED)	0.002	(0.200)	0.032	(0.140)	0.112	(0.208)
Some college or Associate’s degree	0.087	(0.215)	−0.182	(0.155)	−0.310	(0.229)
Bachelor’s degree or above	0.192	(0.272)	−0.327	(0.205)	−0.387	(0.309)
**Household income (ref: 0 to $49,999)**						
$50,000 to $99,999	0.351*	(0.166)	0.138	(0.119)	0.196	(0.170)
$100,000 to $149,999	0.450*	(0.204)	0.235	(0.148)	0.077	(0.215)
$150,000 or more	0.158	(0.174)	0.066	(0.119)	−0.066	(0.178)
**Employment (ref: Working full-time)**						
Working part-time	0.170	(0.164)	−0.059	(0.112)	−0.072	(0.163)
Not working	0.327	(0.175)	−0.104	(0.118)	−0.224	(0.180)
**Region (ref: Northeast)**						
Midwest	−0.243	(0.194)	−0.285	(0.149)	−0.239	(0.219)
South	−0.190	(0.181)	−0.123	(0.132)	0.004	(0.191)
West	−0.100	(0.204)	−0.013	(0.147)	0.043	(0.209)
**Past 30-day cannabis use (ref: No)**						
Yes	−0.027	(0.120)	−0.148	(0.086)	0.010	(0.126)
Observations	486		486		486	

Standard errors in parentheses.

* *p* < 0.05,

** *p* < 0.01,

*** *p* < 0.001.

**Table A8.** Comparison of sociodemographic characteristics between respondents included in the analytical sample (*N* = 820) and respondents with missing BWS responses (“don’t know” or “refused”) (*N* = 190).

Variables	Mean (SD)/Percent		
	Analytical sample	Missing BWS responses	Test difference (P-value)

Age	17.72 (1.69)	17.5 (1.71)	0.108
Gender			
Female	55.92%	48.94%	0.083
Male	44.08%	51.06%	
Race/Ethnicity			
White, non-Hispanic	60.37%	54.21%	0.199
Black, non-Hispanic	10.37%	15.79%	
Other, non-Hispanic	2.80%	4.21%	
Hispanic	18.66%	18.42%	
2+ Races, non-Hispanic	7.80%	7.37%	
Education			
No high school diploma or GED	51.05%	41.46%	0.063
High school graduate (high school diploma or the equivalent GED)	28.42%	28.17%	
Some college or Associate’s degree	18.42%	27.07%	
Bachelor’s degree or above	2.11%	3.29%	
Household income			
Less than $50,000	23.17%	28.95%	0.208
$50,000 to $99,999	30.37%	23.68%	
$100,000 to $149,999	21.22%	22.11%	
$150,000 or more	25.24%	25.26%	
Region			
Northeast	15.98%	17.37%	0.881
Midwest	24.02%	22.63%	
South	37.68%	39.47%	
West	22.32%	20.53%	
Employment			
Working full-time	17.21%	15.30%	0.070
Working part-time	42.34%	34.97%	
Not working	40.45%	49.73%	

## References

[R1] U.S. Census Bureau. (2010). Census regions and divisions of the United States. https://www.census.gov/geographies/reference-maps/2010/geo/2010-census-regions-and-divisions-of-the-united-states.html

[R2] Barrington-Trimis JL, Bae D, Schiff S, Davis J, Unger JB, & Leventhal AM (2020). Susceptibility to cannabis use composite index [Database record]. APA PsycTests. 10.1037/t79118-000

[R3] Bedillion MF, Claus ED, Wemm SE, Fox HC, & Ansell EB (2024). The effects of simultaneous alcohol and cannabis use on subjective drug effects: A narrative review across methodologies. Alcohol, Clinical & Experimental Research, 48(6), 988–999. 10.1111/acer.15322 PMC11238947 38641546

[R4] Birdsey J, Cornelius M, Jamal A, Park-Lee E, Cooper MR, Wang J, Sawdey MD, Cullen KA, & Neff L (2023). Tobacco product use among U.S. middle and high school students—national youth tobacco survey, 2023. Morbidity and Mortality Weekly Report, 72(44), 1173–1182. 10.15585/mmwr.mm7244a1 PMC10629751 37917558

[R5] CDC. (2024). Youth tobacco product use at a 25-year low, yet disparities persist. Retrieved August 28, 2025, from https://www.cdc.gov/media/releases/2024/p1017-youth-tobacco-use.html

[R6] CDC. (2025). YRBS data summary & trends report. Retrieved March 17, 2026, from https://www.cdc.gov/yrbs/dstr/index.html

[R7] Chambers RA, Taylor JR, & Potenza MN (2003). Developmental neurocircuitry of motivation in adolescence: A critical period of addiction vulnerability. The American Journal of Psychiatry, 160(6), 1041–1052. 10.1176/appi.ajp.160.6.1041 PMC2919168 12777258

[R8] Chen-Sankey J, La Capria K, Glasser A, Padon AA, Moran MB, Wagoner KG, Jackson KM, & Berg CJ (2024). Associations between e-cigarette marketing exposure and vaping nicotine and cannabis among U.S. adults, 2021. Addictive Behaviors, 157, 108090. 10.1016/j.addbeh.2024.108090 38880059

[R9] Choi NG, Marti CN, & Choi BY (2024). Associations between Cannabis consumption methods and cannabis risk perception. International Journal of Environmental Research and Public Health, 21(8), 986. 10.3390/ijerph21080986 PMC11353858 39200597

[R10] Cooper M, & Shi Y (2025a). Correct recognition and appeal ratings of copycat cannabis edible packaging: Evidence from an online experiment. Cannabis and Cannabinoid Research, 10(3), 420–424. 10.1089/can.2025.0017 PMC12171700 40354317

[R11] Cooper M, & Shi Y (2025b). Packaging of cannabis edibles, health warning recall, and perceptions among young adults. JAMA Network Open, 8(4), e253117. 10.1001/jamanetworkopen.2025.3117 PMC11969283 40178854

[R12] Donnan J, Johnston K, Coombs M, Najafizada M, & Bishop L (2024). Exploring consumer preferences for cannabis edible products to support public health policy: A discrete choice experiment. PloS One, 19(5), e0292336. 10.1371/journal.pone.0292336 PMC11098505 38753807

[R13] ElSohly MA, Majumdar CG, Chandra S, & Radwan MM (2024). A 10-year trend in cannabis potency (2013–2022) in different geographical regions of the United States of America. Frontiers in Public Health, 12, 1442522. 10.3389/fpubh.2024.1442522 PMC11484017 39421827

[R14] Evans-Polce RJ, Chen B, McCabe SE, & West BT (2024). Longitudinal associations of e-cigarette use with cigarette, marijuana, and other drug use initiation among US adolescents and young adults: Findings from the population assessment of tobacco and health study (Waves 1–6). Drug and Alcohol Dependence, 263, 111402. 10.1016/j.drugalcdep.2024.111402 PMC11577152 39137612

[R15] Evans-Polce RJ, Mongilio JM, McCabe SE, & Veliz PT (2025). Heterogeneity in nicotine, tobacco, and cannabis use among U.S. adolescents and adults aged 12–34 years. American Journal of Preventive Medicine, 69(5), 108033. 10.1016/j.amepre.2025.108033 PMC13188204 40763833

[R16] Farrelly KN, Amini T, Coelho SG, Fox N, Dimitrova N, Hendershot CS, & Wardell JD (2025). Proximal antecedents and acute outcomes of simultaneous alcohol and cannabis use: Systematic review of daily- and event-level studies. Alcohol, Clinical & Experimental Research, 49(1), 43–61. 10.1111/acer.15484 PMC11740185 39552023

[R17] FDA. (2025a). FDA authorizes marketing of 20 ZYN nicotine pouch products after extensive scientific review. FDA. Retrieved August 28, 2025, from https://www.fda.gov/news-events/press-announcements/fda-authorizes-marketing-20-zyn-nicotine-pouch-products-after-extensive-scientific-review

[R18] FDA. (2025b). Results from the annual national youth tobacco survey (NYTS). FDA.

[R19] Fleming CB, Duckworth JC, Rhew IC, Abdallah DA, Guttmannova K, Patrick ME, & Lee CM (2021). Young adult simultaneous alcohol and marijuana use: Between- and within-person associations with negative alcohol-related consequences, mental health, and general health across two-years. Addictive Behaviors, 123, 107079. 10.1016/j.addbeh.2021.107079 PMC8419075 34403871

[R20] Florimbio AR, Walton MA, Coughlin LN, Lin LA, & Bonar EE (2023). Perceived risk of harm for different methods of cannabis consumption: A brief report. Drug and Alcohol Dependence, 251, 110915. 10.1016/j.drugalcdep.2023.110915 PMC11573003 37597308

[R21] Florimbio AR, Walton MA, Duval ER, Bauermeister JA, Young SD, McAfee J, & Bonar EE (2024). Direct and indirect effects of cannabis risk perceptions on cannabis use frequency. Addiction Research & Theory, 32(1), 68–73. 10.1080/16066359.2023.2221029 PMC10805454 38268741

[R22] Flynn TN, Louviere JJ, Peters TJ, & Coast J (2007). Best–worst scaling: What it can do for health care research and how to do it. Journal of Health Economics, 26(1), 171–189. 10.1016/j.jhealeco.2006.04.002 16707175

[R23] Harrison ME, Kanbur N, Canton K, Desai TS, Lim-Reinders S, Groulx C, & Norris ML (2024). Adolescents’ cannabis knowledge and risk perception: A systematic review. The Journal of Adolescent Health: Official Publication of the Society for Adolescent Medicine, 74(3), 402–440. 10.1016/j.jadohealth.2023.09.014 37966406

[R24] Jamal A, Park-Lee E, Birdsey J, West A, Cornelius M, Cooper MR, Cowan H, Wang J, Sawdey MD, Cullen KA, & Navon L (2024). Tobacco product use among middle and high school students—National Youth Tobacco Survey, United States, 2024. Morbidity and Mortality Weekly Report, 73(41), 917–924. 10.15585/mmwr.mm7341a2 PMC11486349 39418216

[R25] Johnston LD, Miech RA, O’Malley PM, Bachman JG, Schulenberg JE, Patrick ME (2022). Monitoring the future national survey results on drug use, 1975–2021: Overview, key findings on adolescent drug use. 10.7302/4142

[R26] Kourgiantakis T, Vicknarajah R, Logan J, Edwards T, Lee E, Craig S, Kaura A, Williams CC, & Marshall S (2024). Understanding youth and young adult cannabis use in Canada post-legalization: A scoping review on a public health issue. Substance Abuse Treatment, Prevention, and Policy, 19(1), 30. 10.1186/s13011-024-00615-9 PMC11184772 38886804

[R27] Lee CM, Calhoun BH, Abdallah DA, Blayney JA, Schultz NR, Brunner M, & Patrick ME (2022). Simultaneous alcohol and marijuana use among young adults: A scoping review of prevalence, patterns, psychosocial correlates, and consequences. Alcohol Research: Current Reviews, 42(1), 08. 10.35946/arcr.v42.1.08 PMC9059839 35548267

[R28] Louviere JJ, Flynn TN, & Marley AAJ (2015). Best-worst scaling: Theory, methods and applications. Cambridge University Press.

[R29] Mullahy J (2015). Multivariate fractional regression estimation of econometric share models. Journal of Econometric Methods, 4(1), 71–100. 10.1515/jem-2012-0006 PMC6075740 30079291

[R30] National Institutes of Health. (2023). Reported drug use among adolescents continued to hold below pre-pandemic levels in 2023 | National Institutes of Health (NIH).

[R31] National Institutes of Health. (2024). Cannabis and hallucinogen use among adults remained at historic highs in 2023 | National Institutes of Health (NIH).

[R32] Pacula RL, Blanchette JG, Lira MC, Smart R, & Naimi TS (2021). Current U.S. state cannabis sales limits allow large doses for use or diversion. American Journal of Preventive Medicine, 60(5), 701–705. 10.1016/j.amepre.2020.11.005 PMC8068584 33589301

[R33] Papke LE, & Wooldridge JM (2008). Panel data methods for fractional response variables with an application to test pass rates. Journal of Econometrics, 145(1–2), 121–133. 10.1016/j.jeconom.2008.05.009

[R34] Patrick ME, Miech RA, Johnston LD, (2024). Monitoring the future panel study annual report: National data on substance use among adults ages 19 to 65, 1976–2023. Institute for Social Research. 10.7826/ISR-UM.06.585140.002.07.0003.2024

[R35] Poyatos L, Pérez-Acevedo AP, Papaseit E, Pérez-Mañá C, Martin S, Hladun O, Siles A, Torrens M, Busardo FP, & Farré M (2020). Oral administration of cannabis and Δ−9-tetrahydrocannabinol (THC) preparations: A systematic review. Medicina (Kaunas, Lithuania), 56(6), 309. 10.3390/medicina56060309 PMC7353904 32585912

[R36] Pulvers K, Jamalian N, Suh E, Faltaoos P, Stewart SL, & Aston ER (2024). Nicotine and cannabis routes of administration and dual use among U.S. young adults who identify as Hispanic, non-Hispanic Black, and non-Hispanic White. Preventive Medicine Reports, 48, 102912. 10.1016/j.pmedr.2024.102912 PMC11550767 39526216

[R37] Romm KF, Dearfield CT, & Berg CJ (2024). Longitudinal patterns of alcohol and cannabis use among U.S. young adults: Correlates and implications for problematic health outcomes. Addictive Behaviors, 158, 108123. 10.1016/j.addbeh.2024.108123 PMC11563046 39127025

[R38] Spear LP (2018). Effects of adolescent alcohol consumption on the brain and behaviour. Nature Reviews. Neuroscience, 19(4), 197–214. 10.1038/nrn.2018.10 29467469

[R39] Spillane TE, Cohen JE, Spindle TR, Thrul J, Moran M, Lee HM, & Giovenco DP (2025). Concurrent availability of cannabinoid and tobacco products in licensed tobacco retailers in three U.S. cities. The International Journal on Drug Policy, 146, 105041. 10.1016/j.drugpo.2025.105041 PMC12707458 41161101

[R40] Spindle TR, Bonn-Miller MO, & Vandrey R (2019). Changing landscape of cannabis: Novel products, formulations, and methods of administration. Current Opinion in Psychology, 30, 98–102. 10.1016/j.copsyc.2019.04.002 PMC7041884 31071592

[R41] Squeglia LM, & Cservenka A (2017). Adolescence and drug use vulnerability: Findings from neuroimaging. Current Opinion in Behavioral Sciences, 13, 164–170. 10.1016/j.cobeha.2016.12.005 PMC5241101 28111629

[R42] Strongin RM, Sharma E, Erythropel HC, El-Hellani A, Kassem NOF, Mikheev VB, Noël A, Peyton DH, & Springer ML (2023). Emerging ENDS products and challenges in tobacco control toxicity research. Tobacco Control, 33(1), 110–115. 10.1136/tobaccocontrol-2022-057268 PMC9758272 35715171

[R43] Sun R, Mendez D, & Warner KE (2022). Use of electronic cigarettes among cannabis-naive adolescents and its association with future cannabis use. JAMA Network Open, 5(7), e2223277. 10.1001/jamanetworkopen.2022.23277 PMC9308048 35867059

[R44] Terry-McElrath YM, & Patrick ME (2018). Simultaneous alcohol and marijuana use among young adult drinkers: Age-specific changes in prevalence from 1977–2016. Alcoholism, Clinical and Experimental Research, 42(11), 2224–2233. 10.1111/acer.13879 PMC6214706 30277588

[R45] Volkow ND, & Boyle M (2018). Neuroscience of addiction: Relevance to prevention and treatment. The American Journal of Psychiatry, 175(8), 729–740. 10.1176/appi.ajp.2018.17101174 29690790

[R46] Waddell JT, McDonald AE, Shah R, & Corbin WR (2023). Daily relations among alcohol and cannabis co-use, simultaneous use, and negative consequences: A day-level latent profile analysis. Cannabis (Albuquerque, N.M.), 6(3), 9–17. 10.26828/cannabis/2023/000171 PMC10683749 38035163

[R47] Xu L, He Y, Park H, Zhang S, Ma S, & Shang C (2026). How tax structures for retail cannabis shape cannabis use among youth and young adults: Evidence from a volumetric choice experiment. The European Journal of Health Economics: HEPAC: Health Economics in Prevention and Care, 27(6), 1191–1212. 10.1007/s10198-025-01875-3 PMC12922899 41460273

[R48] Xu L, Zhang S, & Shang C (2026). How Cannabis Advertising and Price-Based Sales Promotion Shape Early Use: A National Study of Adolescents and Young Adults. Substance Use & Misuse, 1–10. 10.1080/10826084.2026.2670629 PMC13644041 42152556

[R49] Zhu B, Guo H, Cao Y, An R, & Shi Y (2021). Perceived importance of factors in cannabis purchase decisions: A best-worst scaling experiment. The International Journal on Drug Policy, 91, 102793. 10.1016/j.drugpo.2020.102793 PMC7704653 32482489

[R50] Zhu Y, Trangenstein PJ, & Kerr WC (2025). Does cannabis substitute or complement alcohol after recreational cannabis legalization in the Washington State? A three-level mixed-effects modeling. Addictive Behaviors, 162, 108218. 10.1016/j.addbeh.2024.108218 PMC11725433 39644760

